# Diphtheria

**Published:** 1878-10

**Authors:** A. B. Tadlock

**Affiliations:** President


### DIPHTHERIA.

### AN ESSAY READ BEFORE THE KNOX COUNTY (TENN.) MEDICAL SOCIETY, REGULAR MEETING, OCTOBER 10TH, 1878.

By A. B. TADLOCK, A.M. M.D., President.

The neophyte tamer, in the handling, soon becomes conscious of a mule's identity, and connoisseurs all agree as to the specific character of his muleship. He is known to be a hybrid, and neither the doctrine of evolution, nor that of spontaneous generation is required to prove that he is a mule. Not so with diphtheria; differences are rife even among experts as to its nature, and applied science fails to prove (to the satisfaction of all) whether the poison is a *tertiam quid*, a germinal species, or a venomous breath from an enraged and violated Aeolus;—a miasm, a germ, or a cross.

Judging from the tenacity with which *some theorists* hold their *unproven opinions* to be facts, as a psychological experiment, more might be accomplished by investigating their asinine relationship than science has done as yet to explain the vital properties or mysterious force of dipthería poison. I mean no reflection upon the frailties of science, but all praise to all laudable or successful efforts. Differences of learned doctors, as to whether there ever existed such a disease, meet us at the very threshold of our investigations, and first admonish us to pause and take evidence. The burden of proof to establish the affirmative does not require *evolution* to transform the malum Egypticum or ulcus Assyriacum into a new type; nor is it necessary for the more orthodox creed of spontaniety to show that there has been a new generation; for the *advantages* afforded in the defects of diagnosis and the errors of classification offer to both schools a convenient refuge —the one arguing that the disease always existed, but has, up to the present day, been confounded with other diseases; the other claiming that it was recognized by the earliest authorities, and is identically the same in nature, symptoms and pathology, only under a different name.

Skepticism examines the infant carefully as to its personal kinsfolk, its rapid development, and triumphant, but dreadful career—leaving in its wake the untold slain of a thousand fields, made the more heinous with the bitter sorrows and wails of countless disconsolate mothers, as graphically described by Forest and Hecker, by Brettonean, Treauseau and Chomel, and by Villa Keal, Fontena, Herrera, Cortesius, Fothergill, and hosts of contemporary but none the less illustrious writers, and then coolly declares it a natural offspring only endowed with eccentricities, and should be christened, after its legitimate ancestors, croup or scarletina, Mary or Joseph, and not "the new-born." I quote only to show what prominence obtains in the belief that diphtheria is not a specific disease. Sir Wm. Jenner, Bart., M.D., F.R.S., Prof. Clin. Med., in University College, London, in a lecture January 16th, 1875, says: "I am now inclined to think, from my further experience, that the two diseases are really identical, that the so-called croup is diphtheria." West, author of "Diseases of Children," treats of it as a "form of croup."

Dunglison, in his work on practice, agrees with Stokes in considering the "diphtherite of Bretonnean" as "secondary croup."

Dr. Jacobi, of New York, concludes that "croup and diphtheria were identical processes," differing in "clinical features."

Dr. Pepper is reported as saying that "it is hazardous in the extreme to assert that those two diseases" (croup and diphtheria) "are radically distinct."

Some contend that diphtheria is nothing more than aphthæ; others, that it is angina in some of its forms. The inability of pathologists to point out a structural difference between the false membrane of croup and diphtheria is one of the strongest arguments offered in support of the negative. Also, that both diseases are observed to date their origin alike from cold and damp, and that albumenuria appears as a symptom of both.

Slade, of Boston, in his prize essay, says diphtheria is regarded by some as a form of scarlet fever, in which the throat affection is unaccompanied by the eruption which naturally characterizes the latter, and favors the opinion, because the two diseases prevail at the same time, frequently in the same region, and even in the same family, and "in some instances in those who have been attacked by diphtheria, a rash very similar to that of scarlatina has been observed."

Dr. Snelling asserts that "there are many cases which, without losing their character of scarlatinal origin, so assume the appearance of diphtheria that it is impossible to distinguish them."

In support of the theory that diphtheria is a disease *sui generis*, most standard works proclaim. Not only authors but a long list of medical writers and practitioners of celebrity and great experience in treating the disease, testify to its being a specific disease. It has had its place in classification in medical records and reports, both military and civil, sanitary and professional, until the whole world has become familiar with it, and learned to recognize its identity. Its differential symptoms and course are graphically represented by clinicians, so as to appear in bold relief. In the fading vista of ancient medical literature, contemporary writers gather glimpses of diphtheria existing anterior even to rational classification. Frequent and fearful epidemics are referred to, as of the same disease, only under many different names; such as that described by Hecker in Holland, in 1337; and, afterwards, in the latter half of the fifteenth century, by P. Forest; and that which carried off thousands of children in Sicily and Naples in the seventeenth century; extending its withering sway all over Europe in the eighteenth century; and, unsatisfied, laying siege to the shores of the new world—(1771, Bard. N. Y.)

Displeased with the old nomenclature, Bretonneau, in the nineteenth century, instead of angina maligna, membranous croup, Garotillo, morbus suffocatavis, gangrenosa, cynanche maligna, etc., christened the disease with the name of "diphtherite," to represent its fearful history, and his experience during the years 1821–6, whose description and name were adopted by the medical elite of France, among them M. Louis, M'Kenzie, Dariot, et al., who concurred in the opinion of the identity of diphtheria as a separate and distinct disease.

The wider extension and perpetuation of the disease over Europe and America during the present century has led to the general adoption of Bretonneau's title slightly anglicised into diphtheria. No course of lectures in any medical college would be complete without one on diphtheria.

Painfully familiar is the name to households throughout the length and breadth of the United States. No croup, no scarlet fever, no aphthæ, no malignant sore throat, but *diphtheria* tells the story of countless graves grass-grown and forgotten, and of fresh graves a day old, all over the land.

It is said that a quarter of the entire population of Albany, New York, suffered during one epidemic, that of 1858–9 (Paine.)

In the terrible epidemics of 1856–'64, at San Francisco, Dr. Tougeaud says: "But few children attacked by it recovered." During 1860–'64, inclusive, Philadelphia buried 2,000 of her children, victims of diphtheria, which would, by late computation, represent 10,000 sick with it.

Of 2,000 negroes on two plantations in Louisiana, 400 died of this disease in one year. But what of yesterday and to-day? According to late reports the nation is scourged with diphtheria from ocean to ocean—here a little and there a little—while yet no great epidemic prevails anywhere. It finds its victims in the country as well as in towns and villages. For the fourth of a century it has visited some one or another of the mountain valleys of East Tennessee, and left numbers of parents childless.

Such being but an imperfect outline of the history of a disease whose identity is so much questioned, great interest would naturally seem to follow in the study of its origin, nature, symptoms and pathology.

The names of writers in support of the affirmative are legion, but their arguments are founded mostly upon clinical evidences.

Differentially, it is claimed that membranous croup appears generally sporadically, while diphtheria generally is epidemic in character. No one claims that croup is contagious. Contagiousness of diphtheria is yet an unsettled question with many.

Membranous croup seldom attacks children under two or over seven years of age; diphtheria spares no age. Membranous croup appears generally as a local affection of the larynx; diphtheria appears on the tonsils, locally, but is a blood disease. Membranous croup tends to spread upwards in the mouth and fauces, while diphtheria spreads downwards in the mouth, larynx and pharynx.

We have noticed the voice and cough in membranous croup were more husky, and appearing earlier among the symptoms, while in diphtheria, besides being later, they are dryer and sharper. In the former disease death takes place by apnœa generally; in the latter by asthenia usually.

Diphtheria differs from scarlatina in that a rash almost always belongs to the latter, and but seldom is seen in the former, and when it is, appears as an erythematous efflorescence, and not so punctated at first as that of scarlatina. The pulse seems higher in scarlatina than in diphtheria. According to Empis, the membrane in scarlatina is interrupted and aphthous-like, and is easily detached; in diphtheria it is continuous and consistent, and not easily detached. In diphtheria the membrane tends to spread to all mucous or abraded surfaces; in scarlatina it is generally limited to the fauces.

*Sequellæ.*—Suppuration of the glands of the neck frequent in scarlatina, scarcely ever in diphtheria; also in the former anasarca is frequent, but never known in the latter. Paralysis seldom follows scarlatina, but is common after diphtheria. In scarlatina arthritis is frequent, but not so in diphtheria. Pericarditis is frequently met with in scarlatina, but never in diphtheria. In scarlatina albumen in the urine is a sequella, while in diphtheria, if it does appear, it is an accompaniment.

Irregular and intermittent pulse, with heart-clot or heart paralysis, followed by sudden death, is characteristic of diphtheria—never of scarlatina. Second attacks are very rare in scarlatina—very common in diphtheria.

The destructive features of aphthæ are so evident that we only will notice the most prominent—excessive acuteness of sensibility and diffusion of inflammation over the buccal surfaces.

The difference between a tunic of membrane over an unbroken surface and an ulcer with loss of substance is sufficient always to diagnose a case of ulcerative angina from one of diphtheria. Nothing is claimed, we believe, in the histology of the membrane as diagnostic. Reindfleisch says "the membrane in croup is never reproduced." We all know how rapidly that of diphtheria is.

As to the properties of the membrane, much diversity of opinion prevails. Some say it is a superficial exudation not involving even the epithelium—others that the croupous membrane "consists of altered epithelial cells, held together by a protean substance." Rokitansky thinks the mucous membrane is never interfered with, while others state that the exudation has been seen to penetrate and blend with the mucous membrane. It has been stated that if croup was diphtheria, evidence of blood poison should appear as usual after death; also, that depressing medicines would not be tolerated so well.

This is begging the question, however, for there is still a division of opinion among the supporters of the negative and affirmative, as to whether either membranous croup or diphtheria is a local or blood disease. The latter view, however, is almost universally adopted, and in systematic works it is now treated under the head of general, and not as formerly, under local diseases.

Bretonneau contended at first that it was purely a local disease, but afterwards admitted that the blood became, in severe cases, secondarily affected. Oertel, compiler for Ziemssen, the latest work published, asserts its local nature, and offers as proof that diphtheritic matter inoculated into other parts of the body, fails to manifest itself in the fauces, as it does in a natural case, and as it should do were the disease primarily a blood poison. By the same process of reasoning, syphilitic poison injected into any other part of the body should manifest its effect upon the glands where it *generally* appears.

The febrile action of asthemic type, alteration in color and consistence of the blood, general tendency of eruption to appear consecutively on different parts of the body, the occurrence of albuminuria with its epidemic and contagious nature, are mentioned as proofs of its being a constitutional disease.

But a negative school again demands proof of its epidemic and contagious nature, contending that it is not epidemic, but of endemic miasmatic origin, and that it is not contagious. "Trousseau steeped a lancet in a freshly removed false membrane and punctured his left arm and tonsils five or six times." Dogs and other animals have been inoculated, and all with negative results. On the other hand the fact of contagiousness has been amply established by the occurrence of actual cases, and by extended experimentation too lengthy for us here to repeat, and non-susceptibility must account for the negative results in like cases and experiments. The general conviction is that it is highly contagious and infectious, but not in the same degree as measles and small pox. I would rather say that a greater proportion of constitutions are capable of resisting or of tolerating the diphtheritic poison without being affected. Upon the same principle one person is easily and often poisoned dreadfully by being near Rhus Toxicodendron, while dozens of others exposed to immediate contact with its leaves and branches escape unharmed. Is not the cutaneous exhibit of rhus poison well-known? and will some savant, in the might of his scientific knowledge, tell whether that is a local or systemic poison, primarily? And why not look to the distilling dews and morning mists laden with ethereal fragrance exhaled from nature's luxuriant fountains of living sweetness for some of our woes, as well as to the mephitic quagmires, "sloughs of despond," cess-pools and sewers, or to the infusorial microzomes of vegetable and animal life? Is it not a plausible hypothesis that canine madness is sometimes produced by the conflicting elements of excitement. Even the smell of a rose has been known to produce violent hysterics. Hay fever may be mentioned as another example. An increase of ozone in the atmosphere is allowed to be concomitant with prevailing diphtheria. But I am not seeking to advance any new theory, only wishing to escape stumbling over those that are so familiar and stale, yet panoplied in the most inexplainable mysteries, even at the risk of my perpetrating something equally ridiculous.

Here we have miasm offered to *unexplain* the essential cause of diphtheria. There we have the unexplainable fungus or cryptogam to confound our knowledge; and lastly, the all present bacteria in its multiple forms, which, like flies as scavengers, come rather as a blessing than as a curse, as has been charged upon their devoted heads. Alas! the good intentions, and presence of the best are sometimes wrongly interpreted.

But, Ebert declares that there would be no diphtheria if it were not for Huiter and Oertel's micrococci. I should think rather that without diphtheria, or its kindred products, there would be no bacterian micrococci, and there would be no septic diphtherial poison, for I believe the septic toxemia in diphtheria is tertiary—a hybrid or mule, if you please—the result of the union of diphtheritic miasm, forsooth, with the products of bacterian or fungoid depredations, and their entering together into the blood. For here is observed clinically a complete change in symptoms, the febrile condition being replaced rapidly by the asthenic. Even the bacteria under the new state, as if verifying the evolution theory, change rapidly from the micrococci to bacterium termo, spirilum tenue, and the various unclassified forms which they are now known to assume. But a possibility of the institution of the psychological experiment suggested in the first part of my paper intimidates luxuriance of thought, and warns me of trespassing on other's pastures, instead of more profitably reviewing some of the *symptoms* and *treatment* of diphtheria.

The special symptoms in diphtheria, and their physiologico-pathological relations furnish an interesting field for thought and investigation. I will notice the swelling of the glands, and croupal symptoms as of the greatest clinical importance in prognosis. All authorities refer to the nasal and laryngeal complications as the most serious; the latter as premonitory of death by suffocation by reason of the extension of the disease into the larynx and trachea; and the former forebodes lymphatic involvement —swelling of glands, blood poison, and death by asthenia. The difference of the parts, anatomically, must be considered to explain different processes—surfaces thickly supplied with muciparous follicles, as the tonsils, uvula, nasal passages and arches, quickly and easily give up their false coverings; not so with surfaces less supplied with these follicles as the vocal cords. Parts profusely furnished with lymphatics, as the nasal, rapidly, thereby, transmit the poison to the neighboring glands causing them to swell—and thus, too, the blood becomes vitiated— while the tonsils, the palatine arches and uvula, which are sparingly supplied with lymphatic vessels, will entertain the poison exudate, (even for the repetition of the membrane,) for a much longer time without danger to the circulating fluid.

The early swelling of the glands (cervical and submaxillary) with pain or buzzing noise in the ear, and impairment of hearing are ominous symptoms, warning us of the involvment of the nasal part, and the extension to the inner suffface of the tympanum through the eustachian tube. We are informed by the best authority of the difficulty of diagnosis in nasal diphtheria. Oertel says: "It is only when the infection appears in the anterior parts of the nasal cavities, near the nostrils, on the septum, and the anterior surfaces of the turbinated bones, and false membranes develop there, that a diagnosis can be positively made by inspection." A patient of mine who died on the seventh day after the first noticed symptom was observed—("thought to be cold and stopping up of nose,") showed no visible exudate 'till the fourth day, when, by extension, it appeared on the posterior edges of the uvula and posterior pillars as a beautiful fringe upon a roseate border. The peculiar croupy sound has been referred to, and so has the very uncommon occurrence of the erythema, resembling so closely scarlet rash that different writers warn us of the danger of making an erroneous diagnosis. This clinic peculiarity furnishes a plausible foundation for belief in the unity of the morbific elements engendering the two diseases.

For want of time we must pass over the subject of paralysis which so often follows in some shape or other the affection.

A prominent symptom of peculiar interest is furnished in the feeble and intermittent pulse of patients apparrently recovering, accompanied by pain at the pit of the stomach (Meigs & Pepper) and blowing systolic sound of tolerably high pitch attending the heart's action—indicative of of heart-clot and great impoverishment of blood. The heart itself, lungs, pleura, kidneys, bowels, brain, spine and peripheral nerves are each liable to injury, which will have their individual concomitant symptoms.

In the treatment of diphtheria almost the whole Materia Medica has been appealed to, and hundreds of remedies have from time to time been proclaimed specifics. No disease shows in its history a wider field of therapeutic experimentation, and in results science has but little advantage of empiricism. Anti-phlogistics have been altogether abandoned. Locally, astringents and caustics are no longer relied upon. Generally and locally jermicide and disinfecting remedies are used, with tonics and stimulants. Chlorinated tincure of iron is generally used by all schools of medicine. Permanganate of potash and salicylic acid have strong advocates; lime in some of its various forms has been highly lauded. The fumes from unslaked lime with vinegar poured over it, inhaled, has been very serviceable in my experience in the nasal and croupal forms.

Whatever be the plan adopted or medicine used, much for success, will depend upon the tact and perseverance in the administration of remedies, and the skill and energy forthcoming in providing for emergencies, in observing symptoms, and guiding cases according to individual tendencies, and the character of their sequellæ.

In reference to sanitation, great efforts are being made, instituted by boards of health, to discover causes.

The idea is prominently stated with plausible proof in the annual report of Michigan Board of Health for the year 1877, that the use of water fouled by barn-yard matter is as certain to produce diphtheria, as that polluted by fecal or sewer poison will furnish typhoid fever.

But I opine that all these opinions founded upon conditions existing only in cities and their environs, and upon stock farms, fail to satisfy the scrutiny of science when policing for causes among mountain cabins, and valley huts void of even a hen roost, yet equally bereft of its darlings by the fell destroyer, diphtheria.

